# Lessons from modular approach to training for minimally invasive aortic valve replacement : implications for training and outcome

**DOI:** 10.1186/1749-8090-10-S1-A292

**Published:** 2015-12-16

**Authors:** Umair Aslam, Joseph George, Pankaj Kumar

**Affiliations:** 1 Cardiothoracic Surgery, Morriston Hospital, Swansea, SA6 6NL, UK

## Background/Introduction

Minimally-invasive Aortic Valve Replacement (mini-AVR) is being increasingly adopted in clinical practice. Training can be a challenge due to the inherent difficulties of limited surgical exposure. We analysed individual trainee experience in our institutional undergoing mini-AVR training within a series of our cases where all AVRs are routinely undertaken by this approach.

## Aims/Objectives

We assessed the impact and outcome on training in mini-AVR in our centre at both the trainee cohort and individual trainee levels. We assessed the complication rates between trainees and consultant surgeon undertaking mini-AVR in the context of challenging surgical access.

## Method

A single consultant surgeon undertook minimally-invasive aortic valve replacement for all isolated first-time aortic valve replacement, without any selection. Operative records and cardiac surgery database of all patients who had undergone mini-AVR between 2006-2015 were retrieved. Patient demographics, premorbid status, operating surgeon, operative details and outcome were evaluated. We constructed learning curves of individual trainees and compared. Part-procedures were defined and enumerated, and individual experience over time was plotted. Risk stratification was also analysed. p < 0.05 was defined to be statistically significant.

## Results

171 mini-AVRs were undertaken between 2006 and 2015. We defined a case as all parts being undertaken by the operator. Mini-AVR was divided into nine component part-procedures including mini-sternotomy, cannulation, aortotomy, decalcification, implantation, aortotomy closure, de-airing and weaning, decannulation and sternotomy closure. 13% of cases (n = 23) were undertaken by trainees. The proportion of part-procedures undertaken by trainees varied between 13% (all part-procedures) to 87% (single part-procedures) of all cases. The learning curve of five trainees with the highest operative numbers were plotted. The learning curve of a single trainee over time was plotted in part-procedures. (Figure 2). Logistic euroSCORE was not significantly different between trainees and consultants, however trainees took longer bypass and cross-clamp times.

## Discussion/Conclusion

We demonstrate detailed learning curves and outcomes comparison in learning minimally-invasive aortic valve replacement. The challenge in mini-AVR is the access - mini-sternotomy and cannulation, which is the focus in our training programme.

